# Assessment of the Bulgarian Wastewater Treatment Plants’ Impact on the Receiving Water Bodies

**DOI:** 10.3390/molecules24122274

**Published:** 2019-06-18

**Authors:** Galina Yotova, Svetlana Lazarova, Błażej Kudłak, Boika Zlateva, Veronika Mihaylova, Monika Wieczerzak, Tony Venelinov, Stefan Tsakovski

**Affiliations:** 1 Sofia University “St. Kliment Ohridski”, Faculty of Chemistry and Pharmacy, Chair of Analytical Chemistry, 1164 Sofia, Bulgaria; G.Yotova@chem.uni-sofia.bg (G.Y.); zlateva@chem.uni-sofia.bg (B.Z.); v.mihaylova@chem.uni-sofia.bg (V.M.); 2 University of Architecture, Civil Engineering and Geodesy, Faculty of Hydraulic Engineering, Chair of Water Supply, Water and Wastewater Treatment, 1046 Sofia, Bulgaria; ssvetlanalazarova@abv.bg (S.L.); TVenelinov_fhe@uacg.bg (T.V.); 3 Gdańsk University of Technology, Faculty of Chemistry, Department of Analytical Chemistry, 11/12 Naturowicza, 80-952 Gdańsk, Poland; blakudla@pg.edu.pl (B.K.); monwiecz@pg.edu.pl (M.W.)

**Keywords:** wastewater treatment plant, surface water quality, biotests, partial least squares–discriminant analysis

## Abstract

Deterioration of water quality is a major problem world widely according to many international non-governmental organizations (NGO). As one of the European Union (EU) countries, Bulgaria is also obliged by EU legislation to maintain best practices in assessing surface water quality and the efficiency of wastewater treatment processes. For these reasons studies were undertaken to utilize ecotoxicological (Microtox^®^, Phytotoxkit F^TM^, Daphtoxkit F^TM^), instrumental (to determine pH, electrical conductivity (EC), chemical oxygen demand, total suspended solids (TSS), total nitrogen (N) and phosphorus (P), chlorides, sulphates, Cr, Co, Cu, Cd, Ba, V, Mn, Fe, Ni, Zn, Se, Pb), as well as advanced chemometric methods (partial least squares–discriminant analysis (PLS-DA)) in data evaluation to comprehensively assess wastewater treatment plants’ (WWTPs) effluents and surface waters quality around 21 major Bulgarian cities. The PLS-DA classification model for the physicochemical parameters gave excellent discrimination between WWTP effluents and surface waters with 93.65% correct predictions (with significant contribution of EC, TSS, P, N, Cl, Fe, Zn, and Se). The classification model based on ecotoxicological data identifies the plant test endpoints as having a greater impact on the classification model efficiency than bacterial, or crustaceans’ endpoints studied.

## 1. Introduction

Water is a vital resource for all human activities, e.g., everyday necessities, agriculture, manufacturing, transportation. Despite its importance, water is the most poorly managed resource in the world [1]. According to World Health Organization (WHO), the pollution of water is defined as any deterioration of the physical, chemical or biological parameters that leads to an adverse impact on living organisms in the environment or makes the water resource unsuitable for its intended use. Every time water is used, it acquires contaminants, and its quality decreases. Nearly 80% of the used water is returned into the environment untreated. This increases freshwater scarcity worldwide, since the contaminated water may cause human diseases due to the wide variety of viruses, bacteria, and protozoa, these waters may contain. Apart from these biological contaminants, the wastewater effluents are also polluted with chemicals, e.g., nitrogen, phosphorus, heavy metals [2], and organic compounds [3,4] among which detergents, pesticides, hydrocarbons, and metabolites. Wastewater effluents rich in decomposable organic matter are the primary cause of organic pollution. Most heavy metals present in the water are associated with industrial discharges but are also found in the wastewater treatment plants’ (WWTP) effluents [2]. Therefore, the management and utilization of natural resources need to be further improved, and human pollution activities to be reduced. As a result, several legislation documents and guidelines have been developed—the WHO Guidelines for the reuse of effluents (developed in 1973, revised in 1989 and 2006) [5], the UN General Assembly’s Millennium Development Goal for ensuring environment sustainability (adopted 2000) [6], the Water Framework Directive (WFD, adopted in 2000) [7] and its sub-directives 91/271/EEC concerning urban wastewater treatment which establishes requirements for discharges from urban WWTPs [8] and 98/83/EC concerning the quality required of drinking water [9].

The WFD focuses on the effectiveness and sustainability of the water environment through an integrated and coordinated approach to water management [10]. The introduction of the Directive in 2000 had the sole purpose of establishing a framework for the protection of European waters and for the Member States to reach “good status” for water bodies throughout the European Union (EU). Being the first European Directive that focused on environmental sustainability [11,12], WFD was considered as a pilot for future environmental regulations [13], but surface water bodies in “good” state only increased by 10% from 2009 to 2015 [14].

Surface water quality is directly connected with the development of societies. Political changes in post-communistic countries result in changes in the water quality due to the transition from the planned to market economy in 1990. Political and social changes in Bulgaria prior to its accession to the European Union, necessitated new national regulatory requirements, especially in the field of environmental preservation and public health. Harmonization of some laws and national regulations, especially in the field of environment protection, has required state regulations for wastewater discharge (2002) in water bodies, for drinking water quality (2007), and for surface water quality for drinking water supply (2002) to fully comply to the EU directives [8,9,15]. At the beginning of economic changes, pollution decreased due to the transformation of the industry from unprofitable manufactures and the introduction of environmentally friendly technologies to new not so well-developed economic sectors.

The application of multivariate statistical analysis is widely used in environmental pollution assessment studies of different environmental compartments. The use of multivariate statistical techniques enables the interpretation of complex data matrices for a better understanding and assessment of air [16], water [17], soil [18], and sediment quality [19] of the investigated region. Usually, water quality assessment is based on monitoring of water quality indicators at different sampling sites in the respective water body during different seasons [20]. Such monitoring programs generate a large amount of data with a complex structure and “hidden” knowledge concerning water quality. In many water quality assessment studies, multivariate statistical approaches are applied to retrieve important information concerning water quality management such as: (i) outlining similarity groups between water quality indicators and sampling sites [21,22,23,24,25]; (ii) identification of factors (sources) controlling water quality [17,21,23,24,25,26,27]; and (iii) revealing of spatial–temporal variations in the investigated water body [20,21,22,27,28]. Additionally, multivariate statistical results could be used for the optimization of water quality monitoring programs by revealing existing patterns of water quality indicators and sampling sites. Two groups of multivariate statistical techniques for pattern recognition, unsupervised and supervised, are used in water quality assessment studies. The unsupervised methods, such as cluster analysis (CA), principal component analysis (PCA), self-organizing maps (SOM), search for similarity in the monitoring data set without a priori information concerning sample origin. CA identifies groups of similarity between sampling sites with different water quality indicator profiles [21,22,23]. In water monitoring studies, PCA is used for the identification of “hidden” sources controlling water quality [17,21,23,26,27] while application of SOM enables simultaneous visualization of similarity groups among water quality indicators and sampling sites [24,25]. An additional advantage of the SOM application in water quality assessment is the possibility for inclusion of expert information in the data analysis followed by decision support techniques, such as Hasse diagram [29,30,31]. The most widely used supervised pattern recognition method in water quality studies is discriminant analysis (DA). DA is used to determine the water quality parameters, which discriminate two or more predefined groups of sampling sites. DA has been reported as an effective tool to evaluate temporal and spatial changes in water quality [21,22,27,28]. Further, DA helps in the verification of CA results by identification of the discriminating quality parameters between identified groups [23,32].

Among traditionally used CA, PCA, and DA, in the study of Singh et al. [17] partial least squares–discriminant analysis (PLS-DA) has been reported as a potentially valuable statistical tool for water quality assessment. PLS-DA is a widely used method outside environmental assessment studies. The method is predominant in metabolomics [33] and also very popular in other fields where multivariate data need to be evaluated [34]. The PLS-DA method encompasses two steps: (i) PLS components construction; and (ii) discriminant analysis based on extracted PLS components. The approach uses dimensionality reduction and diagnostic capabilities of PLS, which may be useful to represent water quality factors and to outline the importance of water quality parameters. In the second step, discrimination between groups of sampling sites could be performed based on water quality factors as each group is characterized by a concentration profile reflecting its own water quality.

Environmental risk assessment of WWTPs is usually performed by monitoring the quality of WWTP effluents [35,36] or by taking samples from water body receiving treated wastewaters [17,37]. The first approach is preferred when WWTP effluent quality is compared with a water quality guideline and/or WWTP efficiency assessment is performed. The second approach is focused on the impact of WWTP on the surface waters and is more environmentally relevant. Nevertheless, monitoring of the receiving water body itself without quality information for released treated wastewaters could lead to biased assessment provoked by different pollution sources.

The choice of water quality indicators for monitoring is another important issue in water quality assessment. Next to the legislation introduced physicochemical indicators, biotests have proved to be an effective WWTP assessment tool taking into account the combined effect of environmental pollutants and holistic impact on the environmental compartments [37,38]. Further increased consumption of new pharmaceuticals and personal care products lead to the release of new and emerging pollutants, which could be missed by classical instrumental methods. Thus, for the adequate estimation of WWTP’s environmental impact, the introduction in a monitoring scheme of a selected battery of biotests using species from different trophic levels is of particular importance.

The present study aims to assess the impact of the Bulgarian WWTPs on receiving water bodies by (i) collecting samples from WWTP effluents and water bodies receiving treated wastewaters; (ii) monitoring a representative set of physicochemical water quality parameters and biotests with species from different trophic levels; (iii) discriminating water quality factors and parameters between WWTP effluents and receiving water bodies by PLS-DA. To the best of our knowledge, the proposed monitoring scheme, and statistical modeling to evaluate WWTPs’ impact on receiving water bodies are undertaken for the first time in this study.

## 2. Results

### 2.1. Sampling and Basic Statistics

**Figure 1:**
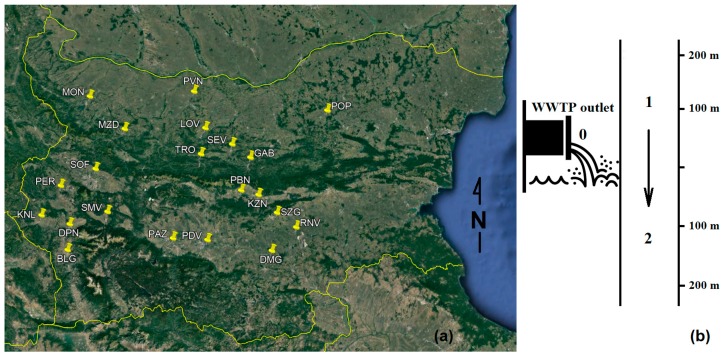
Sampling: (**a**) location of the wastewater treatment plants (WWTPs) and (**b**) sampling scheme (please refer to Supplementary Materials for details on sampling locations and acronyms used).

Sewage water samples were collected from twenty-one Bulgarian WWTPs receiving urban wastewaters (refer to Figure 1 for sampling locations) and from the respective receiving bodies. Samples were taken at three points in case of every WWTP: from WWTP effluents (marked with 0), from the receiving river in the hydrologic course prior to WWTP outlet (marked with 1) and from the watercourse after the release of treated wastewaters (marked with 2).

Eight physicochemical indicators—pH, electrical conductivity (EC), chemical oxygen demand (COD), total suspended solids (TSS), total phosphorus (P), total bound nitrogen (N), chlorides (Cl) and sulfates (SO4)—were determined by spectrophotometric methods using cuvette tests. The concentrations of twelve potentially toxic elements–Cr, Co, Cu, Cd, Ba, V, Mn, Fe, Ni, Zn, Se, Pb—were measured using inductively coupled plasma mass spectrometer (ICP-MS).

Additionally, eight ecotoxicity indicators were included in the data set: percentage inhibition of seed germination (SG)/root growth (RG) of *Sorghum saccharatum* (SS-SG/SS-RG, respectively), *Lepidium sativum* (LS-SG/LS-RG), *Sinapis alba* (SA-SG/SA-RG), percentage bioluminescence change of *Vibrio fischeri* (Microtox) and percentage mortality of *Daphnia magna* (Daphnia).

Altogether, the obtained data matrix consisted of 63 objects and 28 water quality parameters. The basic statistics of water quality parameters for WWTP effluents is presented in Table 1.

As can be seen based on data presented in Table 1, the requirements of Directive 91/271/EEC [8] for the discharges are met for a vast majority of the samples regarding the controlled parameters - COD, TSS, N, and P. For Pazardjik (PAZ) WWTP the concentrations of total nitrogen and total phosphorus are above the concentration limits set in the Directive. Additionally, four results for total phosphorus are higher than the concentration limits–the outlets of Pernik (PER), Plovdiv (PDV), Gabrovo (GAB), and Popovo (POP). These results correspond well with the data obtained from the mandatory monitoring of the studied WWTPs for the period 2015 to 2017 (see Table S2 in Supplementary Materials). In the detailed 3 years’ mandatory monitoring, problems with samples that do not comply with the Directive limits are observed for the same WWTPs for the same parameters—total nitrogen (for PAZ) and total phosphorus (for PAZ, PDV, and POP).

The basic statistics of water quality parameters in surface waters are shown in Table 2.

Directive 75/440/EEC [15] characterizes the possible drinking water sources as Categories A1: needing simple physical treatment and disinfection; A2: requiring normal physical, chemical treatment, and disinfection and A3: with intensive physical, chemical treatment and extended disinfection. The directive sets the limits for quality requirements of surface waters. As can be noticed, based on data presented in Table 2, the water quality parameters in the effluent waters of the studied WWTPs would meet the requirements of the directive if these water bodies were to be used as a source for drinking water abstraction. Thirteen (out of 20) physicochemical parameters studied (refer to Table 2) indicate the water in the rivers could possibly be used as category A1 water for drinking purposes. For nitrogen, the regulations are met only for category A3, for manganese and iron they meet categories A2 and A3.

### 2.2. PLS-DA Models

**Figure 2:**
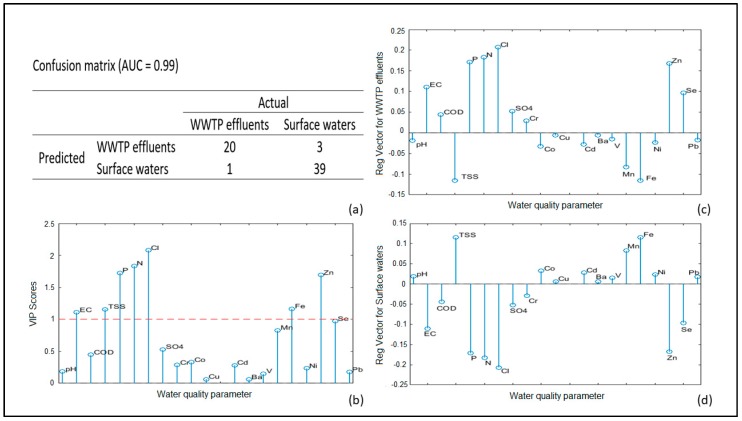
The partial least squares–discriminant analysis (PLS-DA) model results for WWTP effluents and surface waters based on physicochemical water quality parameters: (**a**) Confusion matrix; (**b**) VIP (variable importance on projection) scores; (**c**) Regression vector for WWTP effluents; (**d**) Regression vector for surface waters.

The first PLS-DA classification model was developed for the 20 physicochemical parameters to discriminate samples divided into two classes: WWTP effluents (21 samples) and surface waters (42 samples). The confusion matrix and area under the curve (AUC) value (Figure 2a) resemble the excellent prediction model ability [39] with 93.65% correct predictions.

With significant contribution for the classification model, the following quality parameters could be outlined: EC, TSS, P, N, Cl, Fe, Zn, and Se; their VIP (variable importance on projection) score values are close to or higher than one (Figure 2b). The presented regression vectors for WWTP effluents (Figure 2c) and surface waters (Figure 2d) resembles the concentration profiles of both classes. Concerning the abovementioned significant water quality indicators, the WWTP influents are characterized with higher electrical conductivity and higher concentrations of P, N, Cl, Zn, and Se while the surface waters possess higher levels of TSS and Fe.

There are four misclassified samples. The effluent of the smallest WWTP in this study Pavel banya (PBN) is classified as a surface water and three surface waters samples, two in Kyustendil (KNL) and the one after the WWTP outlet in Stara Zagora (SZG), are classified as WWTP effluents. The reason for wrong surface waters’ predictions could be found in an unauthorized discharge in the corresponding river areas.

**Figure 3:**
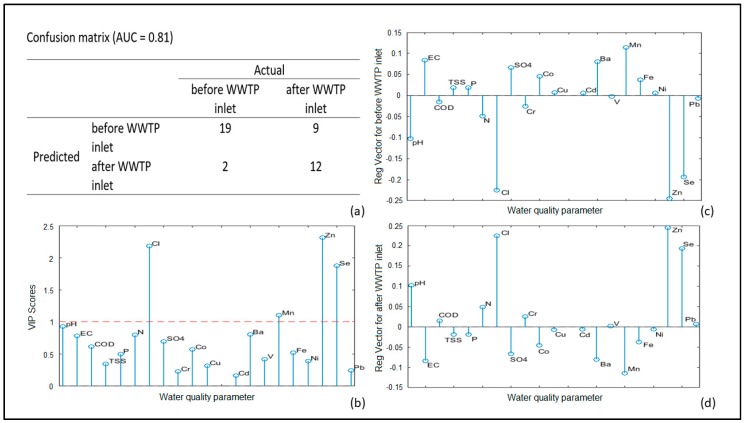
The PLS-DA model results for surface waters before and after WWTP outlet based on physicochemical water quality parameters: (**a**) Confusion matrix; (**b**) VIP scores; (**c**) Regression vectors for WWTP effluents; (**d**) Regression vector for surface waters.

The second PLS-DA model was carried out using the physicochemical indicators to discriminate surface water samples. The surface water samples were arranged in two classes, before and after the WWTP outlet, and each group contains 21 samples. In Figure 3, the results of this analysis are presented.

The AUC value (0.81) corresponds to good accuracy of the classification model. In total, 11 out of 42 samples were misclassified. Two of them (taken before WWTP outlet) are misclassified as samples taken after the release of treated wastewaters (Figure 3a). The sample taken before WWTP outlet of Pernik (PER) has high pH, Cl, and Se levels while the surface water sample before WWTP outlets of Lovech (LOV) possesses elevated pH and Se concentration. The other nine misclassified samples taken after the WWTP outlet are an indication that the released treated wastewaters do not substantially affect the surface water quality in the respected received water bodies. Significant water quality parameters for the classification model are pH, Cl, Mn, Zn, and Se (Figure 3b). The class of samples taken after the WWTP outlet is characterized with higher values of pH, Cl, Zn, and Se, which could be considered as an effect of WWTP discharge on the receiving water bodies (Figure 3d).

**Figure 4:**
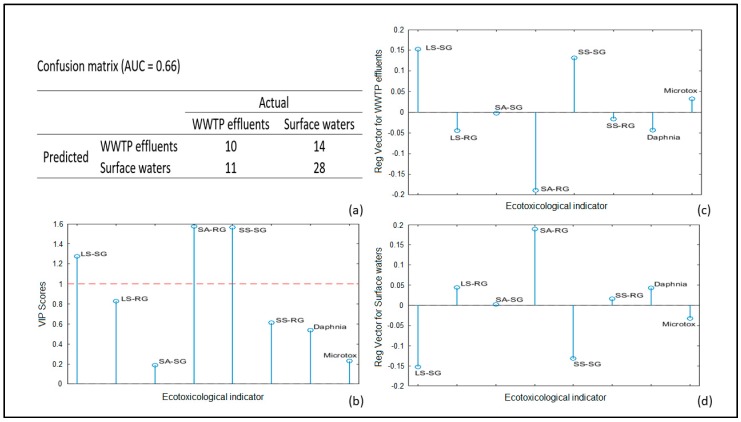
The PLS-DA model results for WWTP effluents and surface waters based on ecotoxicological indicators: (**a**) Confusion matrix; (**b**) VIP scores; (**c**) Regression vector for WWTP effluents; (**d**) Regression vector for surface waters.

Finally, the PLS-DA classification model was developed for eight ecotoxicological parameters to discriminate WWTP effluent samples (21) from the surface waters (42). Although the classification model is not as reliable (Figure 4a) as the previous one (based on physicochemical parameters), some important conclusions could be drawn. The ecotoxicological test Phytotoxkit has a more significant impact on the classification model than Microtox and Daphtoxkit (Figure 4b). The different plant species used in Phytotoxkit give different responses when exposed to WWTP effluents and surface water samples (Figure 4c,d). The numbers of the germinated seeds of *Sorghum saccharatum* (SS-SG) and *Lepidium sativum* (LS-SG) decrease in WWTP effluent samples, whereas the root growth of *Sinapis alba* (SA-RG) increase.

## 3. Discussion

The results obtained in the current study are in good agreement with similar European studies for WWTPs effluents [35] and receiving water bodies subject to WWTP discharge [37]. The water quality parameters in the effluents indicate problems for WWTPs near Pazardzhik (PZK) and Plovdiv (PDV). This may be due to the fact that these treatment plants do not use chemical precipitation of phosphorus and biological nitrogen in the removal facilities. Problems at the outlets of POP, PER, and GAB may be appearing because of the different sampling regimes between mandatory monitoring presented in Table S2 (24 h representative sample) and random sampling used in the current study.

Results for 70% of the studied parameters in the surface waters show good ecological status of the water bodies, and they can be used as category A1 drinking water sources. For 30% of the parameters, surface waters need treatment to achieve the limits for category A1. Iron is present at concentration levels above 0.1 mg/L in all of the samples studied, and content of nitrogen, manganese, phosphorus, and TSS exceeds the respective limits for A1 category in, respectively, 86%, 43%, 19%., and 14% of samples collected.

The excellent discrimination between WWTP effluents and receiving bodies’ surface waters (Figure 2) is based on two groups of significance for the classification model physicochemical parameters. The first one consists of TSS and Fe that has higher concentrations in surface waters than in WWTP effluents. The reason for low concentrations of TSS and Fe in treated wastewaters is the removal of coarse solids found in raw wastewater. The second group of indicators includes P, N, Cl, Fe, Zn, and Se, which possess higher values for WWTP effluents group. The main sources of these elements in wastewaters, excluding toilet loads, are household products used in the bathroom and laundry [40,41].

Discrimination between surface waters taken before and after WWTP outlets is performed to assess the impact of WWTPs on receiving water bodies (Figure 3). The misclassification of nine out of 21 samples taken after the treated wastewater release is an indication that these WWTPs receiving urban wastewaters do not significantly affect the receiving water bodies. The presence of physicochemical parameters with significant impact for 73.81% correct model predictions is due to several reasons. For instance, the higher concentrations of Cl, Zn, and Se in surface waters samples taken after the WWTP outlets are caused by the release of treated wastewaters into receiving water bodies. These water quality indicators could be perceived as a footprint for the WWTPs’ impact on receiving water bodies. Additionally, WWTP discharge leads to an increase of pH and decrease of Mn concentration in surface waters taken after the WWTP outlet.

The lower prediction ability of the classification model based on ecotoxicological parameters (Figure 4) compared to previous PLS-DA models is an indication that some of the used biotests are not applicable for the discrimination of Bulgarian urban WWTP effluents from the receiving water bodies. Daphnia and Microtox tests have no significant contribution to the PLS-DA model. This fact is in agreement with ecotoxicity results for municipality WWTP effluents in Lithuania and Estonia [38]. It seems that these biotests could be effective for ecotoxicity estimation of industrial WWTP effluents. The root growth of all plants used in Phytotoxkit (LS-RG, SA-RG, SS-RG) increase in WWTP effluents as *Sinapis alba* increase has a significant impact on the classification model. This root growth increase in WWTP effluents towards surface waters could be explained with the elevated levels of nutrients (N, P) in treated wastewaters. The other two significant ecotoxicological indicators for classification model is seed germination of *Sorghum saccharatum* (SS-SG) and *Lepidium sativum* (LS-SG). The reason for a smaller number of germinated seeds in WWTP effluents than in surface water samples could be the presence of toxicants in treated wastewaters.

## 4. Materials and Methods

### 4.1. Sampling and Sample Preparation

Sixty-three water samples–21 WWTP outlets and 42 surface waters–were collected in August 2018 according to the scheme described in Section 2.1. Water samples were collected in glass bottles and stored at 4 °C prior to being transported to a laboratory. Fifty milliliters of the sample–intended for ICP-MS analysis–were filtered with a 25 mm PES sterile syringe filters (0.45 µm) and 1.5 mL of concentrated nitric acid was added. Two hundred and fifty milliliters of the sample intended for ecotoxicological analysis was filtered with a 25 mm PES sterile syringe filters (0.2 µm) and frozen.

### 4.2. Physicochemical Analysis

#### 4.2.1. Spectrophotometric Methods Using Cuvette Tests

All steps for sample preparation are described by the producer of the cuvette tests.

The method for the determination of chemical oxygen demand (COD) in water samples using cuvette tests (LCK 314) is based on the oxidation of the sample with potassium dichromate, sulfuric acid, silver sulfate, and mercury sulfate [42]. The solution is heated at 148 ± 2 °C with a thermo-reactor LT 200 (Hach Lange GmbH, Berlin, Germany) for two hours prior to the determination of COD in the range of 15 to 150 mg/L O_2_ using a portable spectrophotometer DR 3900 (Hach Lange GmbH, Berlin, Germany) at 448 nm.

Measurement of total bound nitrogen (N) in water samples with cuvette tests LCK 138 is based on the oxidation of the organic and inorganic forms of nitrogen with peroxydisulphate to nitrates, which then react with 2,6-dimethilphenol in sulfuric acid and phosphoric acid media, yielding nitrophenol [43]. The solution is heated to 100 ± 2 °C (LT 200) for one hour prior to determination of N in the range 1 to 16 mg/L at 370 nm (DR 3900).

The method for the determination of total phosphorus (P) in water samples using LCK 348 is based on the interaction of the phosphate ions with molybdate ions and antimony for the formation of antimonylphosphomolybdate, which is reduced by ascorbic acid to phosphomolybdate blue and heating it for one hour at 100 ± 2 °C (LT 200) prior to determination of P in the range 1 mg/L to 10 mg/L at 890 nm (DR 3900) [44].

Cuvette tests LCK 311 were used for the determination of chloride (Cl) in the range from 1 to 1000 mg/L. The interaction of the chloride ions with mercury thiocyanate produces a release of thiocyanate ions for the formation of iron(III)thiocyanate, and subsequent measurement was performed at 468 nm (DR 3900).

SulfaVer 4 powder reagent was used for the determination of sulfates (SO_4_) in water samples. Sulfate ions in the sample react with barium in the SulfaVer 4 Sulfate Reagent to form insoluble barium sulfate. The measurement in the range from 2 to 70 mg/L was performed at 450 nm (DR 3900).

Measurements of pH and electrical conductivity (EC) were performed on a combined device SensIon+ MM734 (Hach Lange GmbH, Berlin, Germany) [45,46].

The determination method for total suspended solids (TSS) in water is based on the air-pressured filtration of the sample through glass-fiber filters and subsequent drying of the filter at 105 ± 2 °C. The mass of the particles retained onto the filter (1.5 µm) is measured by an analytical balance (RADWAG AC310/C/2, Radom, Poland) with an accuracy of 0.01 g [47].

#### 4.2.2. ICP-MS

Analysis of the water samples was carried out with an ICP-MS PerkinElmer SCIEX - ELAN DRC-e (MDS Inc., Concord, Ontario, Canada). The spectrometer was optimized (RF power, gas flow, lens voltage) to provide minimal values of the ratios CeO^+^/Ce^+^ and Ba^2+^/Ba^+^ as well as maximum intensity of the analytes. External calibration by a multi-element standard solution was performed. The calibration coefficients for all calibration curves were at least 0.99. The measurement conditions for ICP-MS are presented in Table 3.

Single element standard solutions of Ba, Cd, Co, Cr, Cu, Fe, Mn, Ni, Pb, Se, V, Zn (Fluka, Germany) with initial concentration of 10 µg/mL were mixed and used for calibration after appropriate dilution to obtain the following concentrations: 0.5, 1.0, 5.0, 10.0, 25.0, and 50.0 ng/mL. All solutions were prepared with double deionized water (Millipore purification system Synergy, France). For the acidification of the water samples, ultrapure nitric acid (67–69 % HNO_3_, Fisher Chemicals, TraceMetal Grade) was used. The accuracy of the proposed method was checked by analyzing standard reference material NIST 1640a (Trace Elements in Natural Water). The obtained values for analytical recovery varied between 95% and 108%, which was considered as satisfactory.

### 4.3. Ecotoxicological Analysis

To assess the ecotoxicity of the collected samples, a battery of selected biotests was applied. The selected species belong to different trophic levels in the food chain, as follows: producers: *Sorghum saccharatum*, *Lepidium sativum,* and *Sinapis alba* (Phytotoxkit F™, MicroBioTests Inc., Belgium); consumers: *Daphnia magna* (Daphtoxkit F™, MicroBioTests Inc., Belgium), and reducers: *Vibrio fischeri*—Microtox^®^ (ModernWatern, Cambridge, UK).

The Phytotoxkit F™ biotest measures the change of the seed germination and the growth of the young roots after several days of exposure of seeds of selected higher plants to polluted samples, in comparison to a control sample. Originally, this microbiotest is designed to assess the ecotoxicity of soil samples, but Wieczerzak et al. [48] applied it to liquid samples of both environmental and model origin. A layer of cotton wool (100% pure cotton) soaked with the water sample (18 mL) was covered with a filter paper, and 10 seeds of the plant species were placed in the test area. The test was performed in triplicate for each water sample for each one of the three higher plants, and distilled water was used as a control sample. After 72 h incubation at 25 °C, the germinated seeds were counted, images of test plates were taken, and the root growth was measured using the program Image J (NIH, Bethesda, MD, USA) [49].

The biotest Dapthoxkit F^TM^ magna is a crustacean toxicity screening test for freshwater. The test kit contains vials with dormant eggs (ephippia) of *Daphnia magna*. According to the producer’s procedure (MicroBioTests, Inc., Ghent, Belgium), a Standard Freshwater was prepared as hatching and dilution medium. On Day 0 the rinsed ephippia were transferred into a hatching Petri dish in 50 mL pre-aerated Standard Freshwater and incubated at 20 to 22 °C under continuous illumination of minimum 6000 lux for 3 days. The neonates were pre-fed with Spirulina powder 2 h prior to the toxicity test. Each well of the test plate was filled with 10 mL of the samples or Standard Freshwater as a control sample (both in triplicates), and 5 neonates were transferred in each well. The number of dead and immobilized neonates was determined after 48 h incubation in darkness at 20 °C.

The Microtox^®^ biotest utilizes the marine Gram(-) *Vibrio fischeri* bacteria and their ability to bioluminescence. The test Reagent consists of lyophilized bacteria which were rehydrated with a Reconstitution Solution (RS, nontoxic Ultra Pure Water) 20 min prior to the analysis. Osmotic Adjusting Solution (OAS, nontoxic 22% NaCl) was used to adjust the osmotic pressure of the samples to approximately 2% NaCl. The Diluent (nontoxic 2% NaCl solution) was utilized as a control sample and dilution medium. The light emission of the diluted bacteria suspension before and after 30 min exposure to the samples was measured using the Microtox 500 analyzer, and the bioluminescence change was calculated. The data were processed using the Microtox Omni Software, according to the Basic Test Protocol (81.9%).

### 4.4. PLS-DA

PLS-DA is a special form of Partial least square modeling used to find PLS components which discriminate the known classes of samples. The separation between different groups is performed by modeling a relationship between independent input data (**X**) and output data (**Y**). Here the input data (**X**) consists of water quality indicators and (**Y**) is categorical variable (i.e., dummy codes +1 and 0) representing class membership of each sample. For solving of binary classification problems (i.e., two classes) in this study, the PLS1-DA algorithm was performed [34]. Before the analysis, the independent input data (water quality indicators) were autoscaled and venetian blinds as cross-validation procedure was applied. The PLS-DA provides several statistics concerning independent variables (water quality indicators) and sample classes. The variable importance on projection (VIP) is a measure of the importance of variables in the prediction model. Water quality indicators with VIP values higher than 1 are considered to have significant discriminative power in the achieved classification model. The obtained regression vectors represent the variable profile of known sample classes. The main parameters assessing the performance of the prediction model are specificity and sensitivity. Taking the example confusion matrix presented in Table 4 (where Class A is identified as positive: P, and Class B as negative: N), the Class A sensitivity is calculated as TP/(TP+FN) and describes the model ability to classify correctly samples belonging to Class A. The specificity is defined as TN/(FP+TN) and is a measure for the model’s ability to predict membership of samples belonging to Class B. The receiver operator characteristic (ROC) combined both parameters by plotting the sensitivity against 1-specificity for different values of discrimination thresholds. The area under the curve (AUC) is used as the main figure of merit of obtained PLS-DA models. The perfect model prediction corresponds to AUC value equal to 1 since values equal to or lower than 0.5 are an indication for bad classification models [50]. The detailed information on performed PLS-DA models is presented in Supplementary Materials (Table S3).

All PLS-DA modeling calculations were performed in MATLAB R2018b using PLS Toolbox 8.7 (Eigenvector Research Inc, Manson, WA, USA).

## 5. Conclusions

The treated wastewaters of the biggest Bulgarian WWTPs show higher electrical conductivity and higher concentrations of P, N, Cl, Zn, and Se than the receiving surface waters. The WWTPs’ impact on receiving water bodies is characterized by the higher values of pH, Cl, Zn, and Se in surface water samples taken after the WWTP outlet compared to the samples taken before WWTP discharge. The significant impact of the plant tests endpoints on discrimination between WWTP effluents and surface waters proves the potential of such ecotoxicological tests in WWTPs’ impact assessment.

The methodology proposed in the presented study combines original sampling scheme (Figure 1b) and appropriate supervised pattern recognition technique and offers:

- A new way for WWTPs’ impact assessment on receiving water bodies;
- Prioritization of water quality indicators concerning WWTPs’ impact on receiving water bodies;
- Opportunity for selection of optimal water quality indicator set for assessment of WWTPs’ impact.

Additionally, the used methodology is flexible and could include WWTP influent samples to assess not only the WWTPs’ impact but their efficiency as well.

## Figures and Tables

**Table 1:** Basic statistics of water quality parameters in wastewater treatment plant (WWTP) effluents (*n* = 21), concentration requirements for urban WWTP discharges according to Directive 91/271/EEC and number of samples exceeding the Directive.

Parameter	Unit	Mean	Min	Max	St. dev.	Directive 91/271/EEC	Samples Exceeding (n)
pH	-	8.13	7.57	8.51	0.29	-	-
EC	µS/cm	451.7	87.30	1174	275.4	-	-
COD	mg/L O_2_	12.13	5.69	23.40	4.27	125	-
TSS	mg/L	3.26	0.10	9.40	2.19	35/60 ^1^	-
P	mg/L	1.14	<0.50	2.82	0.73	½ ^2^	5
N	mg/L	7.07	1.85	14.20	3.41	10/15 ^2^	1
Cl^−^	mg/L	42.2	17.4	86.6	19.6	-	-
SO_4_^2−^	mg/L	53	5	136	37	-	-
Cr	mg/L	0.0026	0.0005	0.0139	0.0027	-	-
Co	mg/L	0.0002	0.0001	0.0005	0.0001	-	-
Cu	mg/L	0.0023	0.0007	0.0057	0.0015	-	-
Cd	µg/L	0.0113	0.0001	0.0918	0.0270	-	-
Ba	mg/L	0.0272	0.0131	0.0560	0.0132	-	-
V	mg/L	0.0015	0.0006	0.0064	0.0013	-	-
Mn	mg/L	0.0329	0.0043	0.1146	0.0300	-	-
Fe	mg/L	0.239	0.093	0.408	0.085	-	-
Ni	mg/L	0.0028	0.0016	0.0097	0.0018	-	-
Zn	mg/L	0.0222	0.0056	0.0618	0.0162	-	-
Se	mg/L	0.0006	0.0000	0.0026	0.0006	-	-
Pb	mg/L	0.0010	0.0001	0.0144	0.0031	-	-
LS-SG	%	3.02	0.00	6.67	2.27	-	-
LS-RG	%	−30.59	−92.99	33.83	27.54	-	-
SA-SG	%	0.16	−3.45	10.34	4.00	-	-
SA-RG	%	−26.15	−69.55	27.33	25.56	-	-
SS-SG	%	1.31	−3.45	6.90	3.53	-	-
SS-RG	%	14.65	−4.71	40.18	12.58	-	-
Daphnia	%	18.10	0.00	46.67	11.76	-	-
Microtox	%	27.53	−14.36	61.81	18.90	-	-

^1^ The concentrations shown are for more than 10,000 population equivalent (p.e.) and for 2000–10,000 p.e., respectively; ^2^ The concentrations shown are for more than 100,000 p.e. and for 10,000–100,000 p.e., respectively.

**Table 2:** Basic statistics of water quality parameters in surface waters (*n* = 42), guide values for surface water intended for the abstraction of drinking water according to Directive 75/440/EEC and number of samples exceeding the A1 guide values.

						Directive 75/440/EEC			
Parameter	Unit	Mean	Min	Max	St. dev.	A1	A2	A3	Samples Exceeding A1 (n)
pH	-	8.16	7.54	8.53	0.25	6.5–8.5	5.5–9.0	5.5–9.0	1
EC	µS/cm	269.5	26.90	607.0	162.6	1000	1000	1000	-
COD	mg/L O_2_	9.51	<5.00	59.20	9.19	-	-	30	2
TSS	mg/L	10.91	1.80	40.30	9.94	25	-	-	6
P	mg/L	0.38	<0.50	1.37	0.29	0.4	0.7	0.7	8
N	mg/L	2.58	<1.00	9.90	2.01	1	2	3	36
Cl^−^	mg/L	13.0	1.6	39.4	8.2	200	200	200	-
SO_4_^2−^	mg/L	39	7	230	38	150	150	150	-
Cr ^1^	mg/L	0.0021	0.0003	0.0097	0.0025	0.05	0.05	0.05	-
Co	mg/L	0.0003	0.0001	0.0006	0.0001	0.02	-	-	-
Cu	mg/L	0.0024	0.0006	0.0098	0.0019	0.02	0.05	1	-
Cd	µg/L	0.0199	0.0001	0.2256	0.0464	1	1	1	-
Ba ^1^	mg/L	0.0277	0.0067	0.0531	0.0124	0.1	1	1	-
V	mg/L	0.0016	0.0004	0.0043	0.0011	0.01	-	-	-
Mn	mg/L	0.0508	0.0072	0.1182	0.0275	0.05	0.1	1	18
Fe	mg/L	0.388	0.128	0.885	0.186	0.1	1	1	42
Ni	mg/L	0.0031	0.0012	0.0062	0.0012	0.02	-	-	-
Zn	mg/L	0.0069	0.0013	0.0209	0.0045	0.5	1	1	-
Se ^1^	mg/L	0.0003	0.0000	0.0015	0.0003	0.01	0.01	0.01	-
Pb ^1^	mg/L	0.0013	0.0001	0.0068	0.0018	0.05	0.05	0.05	-
LS-SG	%	2.26	0.00	10.00	2.85	-	-	-	-
LS-RG	%	−24.82	−83.91	63.89	33.87	-	-	-	-
SA-SG	%	0.04	−3.45	10.34	3.58	-	-	-	-
SA-RG	%	−16.72	−68.26	27.69	27.61	-	-	-	-
SS-SG	%	0.00	−3.45	10.34	3.73	-	-	-	-
SS-RG	%	13.28	−9.43	45.83	14.99	-	-	-	-
Daphnia	%	19.37	0.00	40.00	9.75	-	-	-	-
Microtox	%	27.29	−16.69	63.41	22.01	-	-	-	-

^1^ The mandatory values, instead of guide values in Directive 75/440/EEC are shown.

**Table 3:** Measurement conditions for ICP-MS (Perkin Elmer SCIEX DRC-e).

Instrument	Operating Conditions
Argon plasma gas flow	15 L/min
Auxiliary gas flow	1.20 L/min
Nebulizer gas flow	0.90 L/min
Lens voltage	6.00 V
ICP RF power	1100 W
Pulse stage voltage	950 V
Dwell time	50 ms
Acquisition mode	Peak hop
Peak pattern	One point per mass at maximum peak
Sweeps/reading	8
Reading/replicates	1
Sample uptake rate	2 mL/min
Number of runs	6
Rinse time	180 s
Rinse solution	3% HNO_3_ (*v*/*v*)
Isotopes monitored	137Ba, 111Cd, 59Co, 52Cr, 63Cu, 57Fe, 55Mn, 60Ni, 208Pb, 78Se, 51V, 66Zn

**Table 4:** Example confusion matrix.

		Actual	
		Class A	Class B
Predicted	Class A	TP	FN
	Class B	FP	TN

## Supplementary Materials

The following are available online, Figure S1: Sampling locations of the WWTPs, Table S1: Sampling locations and acronyms of the WWTPs, Table S2: Number of the samples from the mandatory monitoring of the studied WWTPs for the period 2015 to 2017 exceeding Directive 91/271/EEC, Table S3: PLS-DA models information.
