# Supplementary material for: Assessment of the Bulgarian Wastewater Treatment Plants’ Impact on the Receiving Water Bodies
Source: Molecules. 2019 Jun 18;24(12):2274. doi: 10.3390/molecules24122274 (PMC6630423; doi:10.3390/molecules24122274)
Supplement: Supplementary file 1 [file molecules-24-02274-s001.pdf]

Electronic Supplementary Material
Article

Assessment of the Bulgarian waste water treatment plants impact on the receiving water bodies

Galina Yotova[1], Svetlana Lazarova[2], Błażej Kudłak[3], Boika Zlateva[1], Veronika Mihaylova[1], Monika Wieczerzak[3], Tony Venelinov[2] and Stefan Tsakovski[1*]

[1] Sofia University "St. Kliment Ohridski", Faculty of Chemistry and Pharmacy, Chair of Analytical Chemistry, 1164 Sofia, Bulgaria, e-mail: G.Yotova@chem.uni-sofia.bg (G.Y.); zlateva@chem.uni-sofia.bg (B.Z.); v.mihaylova@chem.uni-sofia.bg (V.M.)

[2] University of Architecture, Civil Engineering and Geodesy, Faculty of Hydraulic Engineering, Chair of Water Supply, Water and Wastewater Treatment, 1046 Sofia, Bulgaria, e-mail: ssvetlanalazarova@abv.bg (S.L.); TVenelinov_fhe@uacg.bg (T.V.)

[3] Gdańsk University of Technology, Faculty of Chemistry, Department of Analytical Chemistry, 11/12 Naturowicza, 80-952 Gdańsk, Poland, e-mail: blakudla@pg.edu.pl (B.K.); monwiecz@pg.edu.pl (M.W.)

* Correspondence: tsakovski@gmail.com; Tel.: +359-2-8161426

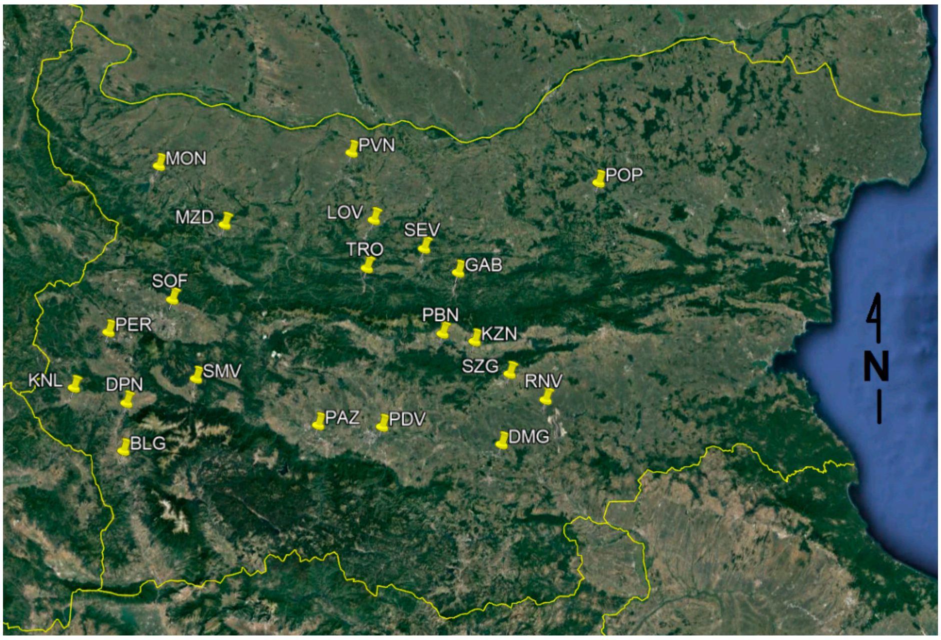

Figure S1. Sampling locations of the WWTPs.

Table S1. Sampling locations and acronyms of the WWTPs

acronym	sampling location	acronym	sampling location	acronym	sampling location
BLG	Blagoevgrad	MZD	Mezdra	POP	Popovo
DMG	Dimitrovgrad	MON	Montana	RDN	Radnevo
DPN	Dupnitsa	PBN	Pavel Banya	SMK	Samokov
GAB	Gabrovo	PAZ	Pazardzhik	SEV	Sevlievo
KZN	Kazanlak	PER	Pernik	SOF	Sofia
KNL	Kyustendil	PVN	Pleven	SZG	Stara Zagora
LOV	Lovech	PDV	Plovdiv	TRO	Troyan

Table S2. Number of the samples from the mandatory monitoring of the studied WWTPs for the period 2015 – 2017 exceeding Directive 91/271/EEC

WWTP[1]	Population equivalent	2015		2016		2017		Series of samples taken in any year[2]	Maximum permitted number of samples which fail to conform[2]
		N	P	N	P	N	P		
PDV	600000	24	24	24	24	22	22	24	3
PAZ	156000	22	22	24	24	24	24	24	3
PVN	188000	2	-	-	-	-	-	24	3
TRO	80000	2	-	-	-	-	-	24	3
DPN	55240	-	-	-	-	-	2	24	3
BLG	87520	-	6	-	6	-	-	24	3
POP	37000	-	1	-	-	-	-	12	2
KZN	80000	-	2	-	-	-	-	24	3

[1]For all the remaining WWTPs and effluent parameters, no exceedings are observed

[2]According to Directive 91/271/EEC

Table S3. PLS-DA models information

Model	LV[1]	Class	RMSEC[2]	RMSECV[3]	Calibration		Cross validation	
					Sensitivity	Specificity	Sensitivity	Specificity
Physicochemical parameters	1	WWTP effluents	0.2590	0.3011	0.952	0.929	0.905	0.905
		surface waters			0.929	0.952	0.905	0.905
Physicochemical parameters	1	before WWTP outlet	0.4280	0.6283	0.905	0.571	0.762	0.381
		after WWTP outlet			0.571	0.905	0.381	0.762
Ecotoxicological parameters	2	WWTP effluents	0.4522	0.5214	0.476	0.667	0.381	0.595
		surface waters			0.667	0.476	0.595	0.381

[1] LV – number of PLS components.

[2] RMSEC – root mean square error of calibration.

[3] RMSECV - root mean square error of cross validation.
